# Most partial domains in proteins are alignment and annotation artifacts

**DOI:** 10.1186/s13059-015-0656-7

**Published:** 2015-05-15

**Authors:** Deborah A Triant, William R Pearson

**Affiliations:** Department of Biochemistry and Molecular Genetics, University of Virginia, Box 800733, Charlottesville, VA, 22908 USA

## Abstract

**Background:**

Protein domains are commonly used to assess the functional roles and evolutionary relationships of proteins and protein families. Here, we use the Pfam protein family database to examine a set of candidate partial domains. Pfam protein domains are often thought of as evolutionarily indivisible, structurally compact, units from which larger functional proteins are assembled; however, almost 4% of Pfam27 PfamA domains are shorter than 50% of their family model length, suggesting that more than half of the domain is missing at those locations. To better understand the structural nature of partial domains in proteins, we examined 30,961 partial domain regions from 136 domain families contained in a representative subset of PfamA domains (RefProtDom2 or RPD2).

**Results:**

We characterized three types of apparent partial domains: split domains, bounded partials, and unbounded partials. We find that bounded partial domains are over-represented in eukaryotes and in lower quality protein predictions, suggesting that they often result from inaccurate genome assemblies or gene models. We also find that a large percentage of unbounded partial domains produce long alignments, which suggests that their annotation as a partial is an alignment artifact; yet some can be found as partials in other sequence contexts.

**Conclusions:**

Partial domains are largely the result of alignment and annotation artifacts and should be viewed with caution. The presence of partial domain annotations in proteins should raise the concern that the prediction of the protein’s gene may be incomplete. In general, protein domains can be considered the structural building blocks of proteins.

**Electronic supplementary material:**

The online version of this article (doi:10.1186/s13059-015-0656-7) contains supplementary material, which is available to authorized users.

## Background

The discovery of evolutionarily mobile protein domains in the early 1980s, shortly after the recognition of eukaryotic splicing, revolutionized our understanding of protein structure. Before the discovery of the exon-shuffled domains in the EGF receptor [1,2], most proteins (globins, cytochrome c, serine proteases, etc.) were understood to be globally similar single-domain proteins. While proteins like calmodulin were known to contain repeated domains, the structural implications of modular proteins were not fully appreciated until clearly homologous domains were seen in different sequence contexts.

Today, domains are central to our understanding of the structure, evolution, and functional roles of proteins and protein families. Protein domain assignments using Pfam [3], InterPro [4], and other domain annotation resources are widely used to infer protein evolutionary relationships, because it is often the protein domain, rather than the protein as a whole, that is conserved over evolution. Evolutionarily conserved, structurally compact protein domains are often found in very different sequence contexts, and only by subdividing a protein into its constituent domains can one understand its evolutionary history.

Some protein domains have clearly understood functions [5]. For example, protein kinase domains are catalytic modules with well-defined roles; other domains direct protein–protein interactions, target other protein modifications or play critical roles in binding and signal recognition (e.g., SH2, SH3, or EF-hand Ca-binding). Identification of these domains helps identify the biological function of the protein containing them.

The evolutionary, structural, and functional roles of domains suggest that domains are the indivisible building blocks from which larger modular proteins are built. Thus, we were surprised to find that 5% to 10% of protein domain annotations in the Pfam protein domain database suggest that only a fraction of the domain is present in the protein. These partial protein domains can cause problems with iterative profile-based similarity searches [6]. Restricting PSI-BLAST searches to libraries of proteins with full-length Pfam protein domains dramatically reduces position-specific scoring matrix (PSSM) corruption, and improves PSI-BLAST specificity and sensitivity [6]. Because PSSM contamination is often caused by the extension of a homologous alignment into a non-homologous neighboring sequence, alignment to a partial Pfam domain might corrupt a PSSM by nucleating a non-homologous alignment across the part of the domain that was missing from the partial domain location. However, if domains are indivisible then the nature of partial domains is puzzling. Do the boundaries of partial domains correspond to structurally distinct regions, or are they both evolutionarily mobile and structurally diverse? Are these partial domains authentic structural units or possible annotation artifacts?

To investigate the nature of these partial protein domains, we used the Pfam database, which uses hidden Markov models (HMMs) to scan UniProt protein sequences and classify conserved domain regions [3]. Pfam has been widely used to characterize the dynamics of protein domain coverage [7], compare sequence and structure [8], and predict erroneous protein sequences [9]. However, profile HMMs do not always detect full-length domains, even when they are present. Sometimes, only the most conserved part domain aligns with the HMM, leading to annotation errors [10]. To examine in detail a set of protein domains representative of the Pfam database, we used domain annotations from the RPD2 protein database [11] that appeared to include less than 50% of the protein domain family model length.

## Results and discussion

### Protein domain lengths

To characterize partial domains in proteins, we examined 136 domain families from Pfam27 (the RDP2 subset, see Methods). We chose Pfam because it is the largest contributor to the InterPro compendium of protein domain databases (Pfam annotates more than 40 million sequences of the 42 million sequences in UniProt/InterPro; the next most comprehensive annotation source covers about half as many). Pfam provides both a model_length parameter, which can be thought of as the characteristic length of the Pfam domain family, and the model_start and model_end coordinates, which we used to calculate the coverage, or partial-ness of the domain in the sequence.

**Figure 1:**
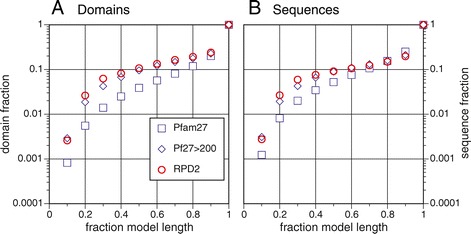
Distribution of partial domain lengths, Pfam27 and RPD2.**(A)** Cumulative fraction of domains versus fractional domain length. Cumulative fractions are shown for Pfam27 domains found in proteins marked as not fragments (24 million domains in total, of which 945,100 are <50% of model length, blue squares) and the RPD2 domains in Pfam27 (290,148 domains, 30,030 <50% of model length, red circles). Also shown are Pfam27 domains from families with more than 200 match states (6.9 million domains, 658,089 <50% partials, blue diamonds). **(B)** Cumulative number of sequences with increasing domain length. Cumulative fractions for Pfam27 sequences (16 million sequences, 820,000 with <50% partials, blue squares) and RPD2 sequences (274,000 total, 27,000 with a domain <50% of model length, red circles). Blue diamonds show sequences containing domains with model length >200 match states (6.3 million sequences, 557,941 <50% partials).

Figure 1 shows the fraction of Pfam27 and RPD2 domains (A) and sequences (B) that contain at least one domain at different fractional coverage of the model_length characteristic domain length. More than 80% of Pfam27 domain mappings cover 90% or more of the domain model_length (Figure 1), consistent with the view that most Pfam domains are discrete-length structurally compact building blocks. Likewise, 75% of the sequences annotated by Pfam contain domains that are ≥90% of the family’s model_length. While very short partial domain instance alignments that cover <20% of model length are quite rare (about 0.5% of all domains and non-fragment sequences in Pfam27), the numbers are large (134,676) because there are more than 24 million Pfam27 domains and 15 million Pfam27 sequences. In this report, we focus on domain annotations where 50% or more of the Pfam HMM, which defines the Pfam family, is missing at the domain annotation on the protein. In the 15 million sequences in Pfam27, there are 945,100 partial domains that are <50% of the model length in 820,720 different sequences. The numbers of partial domains in Figure 1 exclude proteins annotated as fragments; including protein fragments increases the number of <50% partials from 0.95 to 2.0 million.

**Figure 2:**
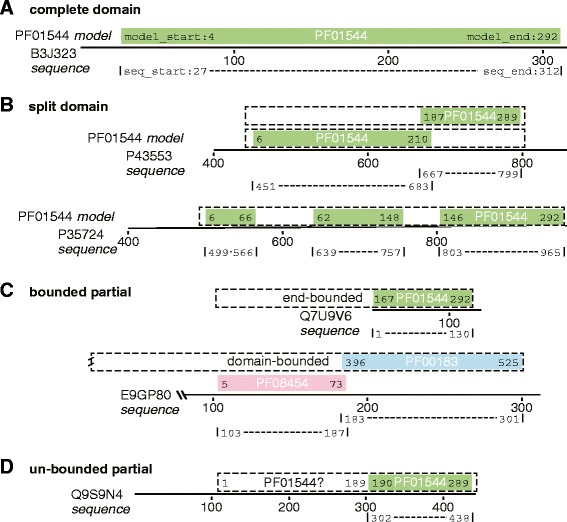
Complete, bounded, and unbounded partial domains. A complete domain, and three types of partial Pfam27 domain mappings. **(A)** Annotation of the complete [Pfam:PF01544] domain in *Bacillus anthracis* CorA protein [UniProt:B3J323]. The full length of the Pfam27 domain is shown in light green, as are the coordinates of the aligned domain in the [Pfam:PF01544] model (model_start, model_end) and [UniProt:B3J323] protein sequence (seq_start, seq_end). **(B)** Split domains. Annotation of [Pfam:PF01544] domains in yeast ALR2 [UniProt:P43553] and MNR2 [UniProt:P35724]. **(C)** Partial domains bounded by the ends of the sequence [UniProt:Q7U9V6] or other domains [UniProt:E9GP80]. **(D)** An unbounded partial domain in [UniProt:Q9S9N4].

In reporting the instances of partial domains in Pfam protein annotations, we distinguish Pfam families – the set of largely distinct protein domains that are associated with different Pfam hidden Markov models (HMMs) [12] – from Pfam domains – the annotation of a domain, or partial domain, at a particular location in a protein. Because a single protein can be annotated to contain multiple Pfam domains from the same family (Figure 2B), we report both the number of Pfam domains (individual HMM mappings to a sequence) and Pfam sequences. When the biological domain has been split into multiple parts by the HMM alignment process (Figure 2B), the Pfam sequence count is a more conservative estimate of the number of partial domains.

While Pfam provides a very comprehensive annotation of domains in proteins, it is difficult to present statistics for representative Pfam families because of the wide range of family model lengths (maximum 2,208, minimum 7, median 134), family sizes (maximum 363,409, minimum 2, median 333), and number of sequences containing a particular Pfam family (maximum 313,128, minimum 1, median 315). Thus, we focused on the RPD2 subset of Pfam27. RPD2 Pfam families have at least 100 members, with no more than 5,000 sequences for any domain. RPD2 also requires that the Pfam family have at least 200 match states; distantly related partial domains with shorter model_lengths can be difficult to detect. RPD2 also limits families from Pfam clans. Pfam clans [12] are used to capture Pfam families that, while homologous, are so evolutionarily diverse that the members of the family cannot be identified with a single HMM. In Pfam27, clan sizes range from 1 to 202 Pfam families (5 clans include more than 100 Pfam families) and different Pfam families in the same clan can have a wide range of model lengths, which greatly complicates the concept of a characteristic domain length. Seventeen Pfam27 clans include families with model lengths that differ at least tenfold. RPD2 only allows one member of a clan, and only if the range of family model_lengths in the clan differs less than twofold. With these restrictions, RPD2 contains only 18 families from clans.

The sample of 136 diverse Pfam27 families in RPD2 shows the same distribution of partial domain model_length as Pfam27 domains with model lengths >200, one of the requirements for RPD2 (Figure 1). Thus, we believe that RPD2 provides a representative sample of domains that are likely to be detected as partials.

### Classification of partial domains

While 80% of RPD2 domains cover more than 90% of the domain model length (Figure 1), 10% of the domains in RPD2 have an annotation that suggests that less than 50% of the domain length is present. Pfam identifies homologous domains in proteins by identifying significant alignments between family HMMs and individual protein sequences. Each HMM:protein sequence alignment defines the start and end of the alignment in the sequence (seq_start and seq_end) and the corresponding boundaries of the domain in the PfamA model for that protein (model_start, model_end). For example (Figure 2A), the CorA-like Mg ^2+^ [Pfam:PF01544] domain in [UniProt:B3J323] is almost full length; all but three match states in the [Pfam:PF01544] model (light green, model length 292 match states) map to [UniProt:B3J323]. A domain can appear to be full length or partial for different reasons, depending on the sequences that bound the candidate partial.

**Figure 3:**
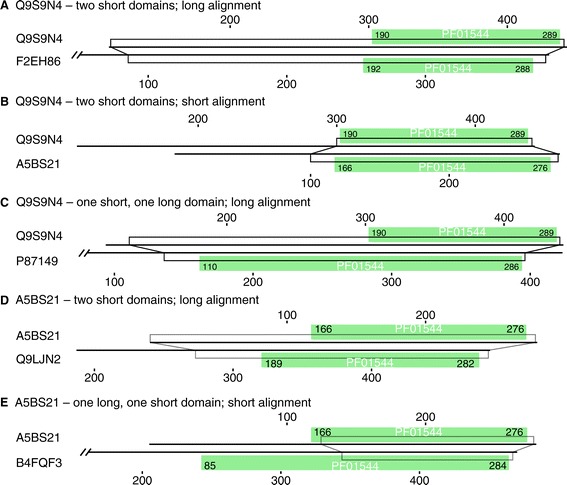
Longer alignments with extended partial domains. Two proteins annotated with partial [Pfam:PF01544] domains, [UniProt:Q9S9N4:112-411] and [UniProt:A5BS21], were compared to the RPD2 proteins using SSEARCH36 (BLOSUM62 scoring matrix, gap open/extend −11/ −1). (A), (B), and (C) show three representative alignments from the 156 RPD2 sequences sharing statistically significant similarity (*E*()<10^−6^) with [UniProt:Q9S9N4] (residues 112 to 411). Lines indicate the protein sequences; open trapezoidal boxes show the projection of the alignments onto the sequences. Shaded boxes map the [Pfam:PF01544] domains annotated on the proteins. The numbers in the shaded boxes report model start and end coordinates from Pfam27. **(A)** One ([UniProt:Q9S9N4:UniProt:F2EH86]) of the 67 longer alignments (>200 amino acids) between proteins with short domain annotations (<200 residues). Non-self-alignments in this category ranged from 26% to 99% identical with 10^−10^<*E*()<10^−157^. **(B)** Alignment of [UniProt:Q9S9N4] with [UniProt:A5BS21], a short alignment (150 residues) between two much longer proteins. **(C)** One alignment ([UniProt:Q9S9N4:UniProt:P87149]) representative of the 86 long alignments (>200 residues, *E*()<10^−6^) to proteins with >50% partial [Pfam:PF01544] domain annotations. **(D)** One ([UniProt:A5BS21:UniProt:Q9LJN2]) of the five non-self-alignments >200 amino acids between proteins with [Pfam:PF01544] domain annotations <200 amino acids (51% identity, *E*()<10^−54^). **(E)** A short alignment (156 residues, 37% identity, *E*()<10^−15^, [UniProt:A5BS21:UniProt:B4FQF3]) where one protein is annotated to contain ≥200 matches to [Pfam:PF01544].

To understand better the computational and biological processes that might produce partial domain annotations, we divided RPD2 candidate partials into three types (Figure 2 and Table 1) based on their sequence context: (1) split domains – single domains that have been broken into several parts by the HMM alignment process (18,624 domains, Figure 2B and Table 1); (2) bounded partials – domains that are bounded by other non-homologous domains or the ends of the protein (5,087 domains, Figure 2C) and (3) unbounded partials – those that appear to be partial but are found in a region of protein that could contain a more complete domain (7,250 domains, Figure 2D).

**Table 1:** **Partial domains in RPD2**

	**Sequences**	**%**	**Domains**	**%**			
Total	270,776	100.0	290,148	100.0			
50% partials	25,116	9.33	30,961	10.72			
Split	13,090	4.84	18,624	6.42			
Bounded	4,953	1.88	5,087	1.80			
Unbounded	7,073	2.62	7,250	2.50			
Putative partial	2,118	0.78	2,156	0.74			
	**Minimum**	**1st quartile**	**Median**	**Mean**	**3rd quartile**	**Maximum**	**PfamA** ^**a**^
Sequences (number)	106.0	515.5	1,544.0	1,991.0	3,323.2	4,918.0	PF00115
50% partials (%)	0.38	2.65	4.68	9.58	11.23	77.82	PF00209
Split	0.00	0.40	1.14	4.76	4.18	72.49	PF00209
Bounded	0.00	0.76	1.31	1.97	2.53	30.51	PF00374
Unbounded	0.00	0.50	1.34	2.84	2.65	38.65	PF04734
Putative partial	0.00	0.18	0.45	0.91	0.89	21.67	PF00852
Domains (number)	113.0	570.2	1,661.5	2,132.4	3,486.2	6,557.0	PF00501
50% partials (%)	0.43	2.72	4.82	9.97	11.54	76.74	PF03069
Split	0.00	0.52	1.48	5.42	4.88	73.97	PF03069
Bounded	0.00	0.73	1.32	1.85	2.34	24.20	PF00374
Unbounded	0.00	0.49	1.30	2.70	2.44	36.40	PF04734
Putative partial	0.00	0.17	0.42	0.86	0.83	21.28	PF00852

^a^The Pfam27 family that produced the maximum percentage of partial domains in the corresponding partial category.

### Split domains

More than half of the candidate <50% partials in RPD2 are parts of longer domain annotations that have been broken into pieces by the local HMM alignment. For example, both yeast ALR2 [UniProt:P43553] and MNR2 [UniProt:P35724] contain <50% partial [Pfam:PF01544] domains, but in both of these cases, the missing part of the domain can be found annotated upstream or downstream (Figure 2B). Thus, a complete domain appears to be present. In ALR2 [UniProt:P43553], the domain from residue 667 to 799 appears to be partial because it includes only 103 of the 292 match states of the [Pfam:PF01544] model, but the ‘missing’ N-terminal two-thirds of the model can be found immediately adjacent to the N-terminal end of the partial domain in the protein.

Likewise, yeast MNR2 [UniProt:P35724] appears to contain three partial instances of the [Pfam:PF01544] domain, but the domain-model mapping suggests that a single complete domain is present. Here, the problem of accurate alignment boundaries can be seen. The center mapping of the [Pfam:PF01544] alignment appears to overlap the N-terminal and C-terminal mappings by several match states, despite the considerable distance between those parts of the domain in the [UniProt:P35724] sequence (Figure 2B). Partial domains that are adjacent to the same domain (or a domain in the same clan) in an orientation consistent with a single larger domain account for about 60% of partial domains (Table 1, Additional file 1: Table S1). Because these alignments are consistent with a single longer domain, we do not consider them partials, and we focus on the domain topologies illustrated in Figure 2C,D.

### Some bounded partials reflect protein annotation artifacts

Bounded partials are limited in length by either another domain, or by the end of the protein sequence (Figure 2C). Bounded partials are unlikely to be artifacts of the HMM alignment process since bounded partial domains cannot be extended past the ends of the protein or into a non-homologous neighboring domain. For example, [UniProt:Q7U9V6], a putative cation transporter from *Synechococcus* sp., is only 141 amino acids long, and thus cannot contain 50% of the 292 match state [Pfam:PF01544] domain (as annotated, it contains match states 167 to 292). Likewise, in [UniProt:E9GP80], a putative uncharacterized protein from *Daphnia pulex*, the [Pfam:PF00183] (Hsp90) domain cannot be extended to include the missing 394 match states because a different non-homologous domain ([Pfam:PF08454], RyR and IP3R homology associated) sets the N-terminal boundary of the partial domain.

Bounded partials may be produced by unusual protein sequence predictions, e.g., alternatively spliced isoforms that do not produce functional proteins, or inaccurate genome assemblies. To look at the relationship between bounded partial domains and protein sequence accuracy, we asked whether bounded partials are enriched over non-partial sequences in organisms that contain introns (eukaryotes), and thus might be splicing or assembly artifacts. We examined the 94 RPD2 Pfam27 families with 10 or more bounded 50% partial domains and asked whether the bounded partial-containing proteins in these families were more likely to come from eukaryotes than RPD2 proteins that do not contain partial domains. If bounded partial domains are due to inaccurate gene models, we expect the errors more frequently in eukaryotes. We performed Fisher’s exact test on each of the 94 families, and then calculated the false-discovery rate (*q* value) to identify families that are significantly enriched for eukaryotic bounded partials (see Methods). When the non-partial and bounded-partial sequence sets were divided into eukaryotic/non-eukaryotic sets, 47 of the 94 bounded partial sets were enriched for eukaryotic sequences at a *q* value (false-discovery rate) of <0.05, and 34 at <0.01. We conclude that many bounded partial domains result from inaccurate gene models that produce incomplete proteins.

In addition, we examined the relative abundance of bounded partial domains from very carefully annotated genomes (human, mouse, and *Drosophila*) in reviewed proteins from SwissProt with an Ensembl gene model. During the Swissprot review process, multiple alternatively spliced transcripts with different accession numbers in the TREMBL division of UniProt are merged into a single accession and labeled as isoforms [13]. Only 4 of the 161 human, mouse, and *Drosophila* proteins (114 from human) in the RPD2 bounded partial category have been reviewed by SwissProt, and one of those does not have an Ensembl gene model. In contrast 848 of 2,893 non-partial RPD2 proteins from human, mouse, and *Drosophila* have been reviewed and 790 have an Ensembl gene model. Bounded partial domain proteins from human, mouse, and *Drosophila* are dramatically enriched in unreviewed sequences lacking Ensembl gene models (*P*<10^−15^, Fisher’s exact test). Since bounded partials are rarely found in carefully annotated full-length proteins from these organisms, we believe that many are likely to be incomplete splice isoforms or other annotation artifacts.

### Unbounded partials

Excluding split domains, a majority of the candidate partial domains belong to the unbounded partial category (Figure 2D). These domains are annotated as partial, but could contain a full-length domain, because there is room for the missing part of the domain in the sequence. Thus, if the [Pfam:PF01544] HMM is projected onto the [UniProt:Q9S9N4] sequence from Figure 2D, one obtains a sequence starting at residue 112, or 330 residues that could map to the 292 match state [Pfam:PF01544] model. When that sequence [UniProt:Q9S9N4:112-] is compared to the sequences in RPD2 Pfam27 using SSEARCH with Blosum62, 156 of the 4,760 sequences containing [Pfam:PF01544] in RPD2 have *E*()<10^−6^, and all of those alignments contain an annotated [Pfam:PF01544] domain. However, all but two of the 10^−6^ homologs align over more than 200 amino acids, and more than 75% align over more than 250 residues, close to the 292 match states in the [Pfam:PF01544] model. The three major types of alignments are shown in Figure 3. In about 40% of the homologs (67/156), the alignments are long, but the domains annotated on the proteins are much shorter (Figure 3A); these proteins are most closely related to the [UniProt:Q9S9N4] query. For most of the more distant homologs (86/156), the alignment is still long, but the aligned sequence is also annotated as containing a full-length [Pfam:PF01544] domain (Figure 3C). In two cases ([UniProt:A5BS21], *E*()<10^−16^, Figure 3D,E and [UniProt:B7FG10], *E*()<10^−20^, not shown), both the annotated domains and the alignments are short. For the [UniProt:B7FG10] alignment, the alignment is short because the protein is short and contains a bounded partial.

If [Pfam:PF01544] contains a true partial domain that can be found in different protein contexts, then we expect that the short alignment with [UniProt:A5BS21], seen in Figure 3B, would reflect novel sequence context, rather than incomplete alignment. A search with the full [UniProt:A5BS21] sequence suggests that [UniProt:A5BS21] does contain an evolutionarily mobile sub-domain. Most of the [UniProt:A5BS21] alignments involve partial domains that produce short alignments like Figure 3B (alignments with 10^−25^ < *E*() < 10^−6^ and 29% to 48% identity), but [UniProt:A5BS21] sometimes produces long alignments with more closely related sequences that are annotated as having partial [Pfam:PF01544] domains (Figure 3D, five non-self-alignments with 10^−54^<*E*()<10^−39^, 44% to 51% identity). Moreover, sometimes [UniProt:A5BS21] produces short alignments with distantly related proteins with longer [Pfam:PF01544] domains (Figure 3E). While many short alignments and partial [Pfam:PF01544] annotations reflect incomplete alignments caused by long evolutionary distances, the instances of short alignments at modest evolutionary distances (>40% identity) suggest that the C-terminal half of [Pfam:PF01544] contains an evolutionarily mobile sub-domain. Below, we show that part of [Pfam:PF01544] is homologous to a smaller CATH structural domain.

### Putative partials

To identify candidate true partial domains from the set of unbounded partials (Figure 2D), we generated the projected full-length domain regions from candidate unbounded proteins and compared the candidate full-length domains to RPD2 proteins. Using the logic described above for [Pfam:PF01544] partials, we sought examples like [UniProt:A5BS21] that have some long domain homologs but also many short domain homologs. Putative partial domains met two criteria: (a) the extended query found ten or more homologs with *E*()<10^−6^ and (b) at least 25% of the homolog alignments were <50% of the family model length. These Pfam families are counted as putative partials in Table 1. In total, 48 of the RPD2 families had more than 10 queries that met these criteria (22 had more than 25 queries). These extended queries that met criteria (a) and (b) were then compared to sequences with known structures to identify putative partial domains.

**Figure 4:**
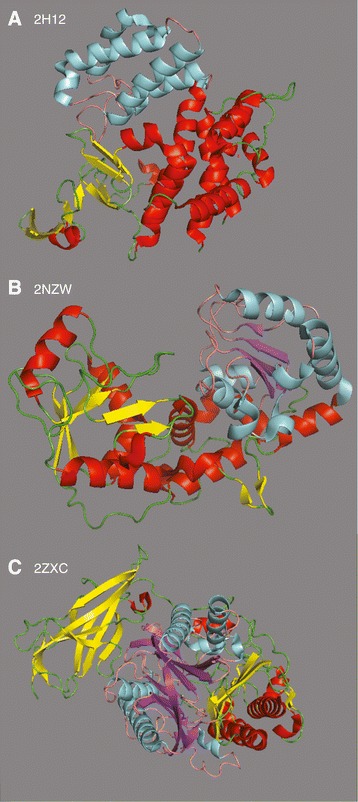
Structural partials.**(A)** The structure of [PDB:1H12] with secondary structures for CATH domain 1.10.580.10 (58 to 274, 385 to 410) highlighted in red (helix) and yellow (strand), and secondary structures for the CATH domain 1.10.230.10 (275 to 384) that aligns with the partial [Pfam:PF00285] region of [UniProt:Q98FC2], highlighted in cyan (helix) and salmon (coil). **(B)** The structure of 2NZW, the most abundant putative partial ([Pfam:PF00852]), with residues 180 to 330 highlighted. **(C)** The structure of 2ZXC, a representative of the PF04734 PfamA family. The putative partial analysis identifies a shorter alignment in the first 200 residues of the PfamA family, and VAST+ annotates a domain from residues 1 to 292, which are highlighted.

About one quarter of our candidate partials map to the Pfam model in a way that leaves room for a much more complete Pfam domain (unbounded partial, Figure 2D). Searches with those extended sequences show that most of them produce long alignments. Thus, those domain annotations are partial because of the inability of the Pfam model to capture the entire homologous region for very distant domain homologs (Figure 3). However, about 30% of these extended sequences produced short alignments, suggesting an alternative sequence context (putative partials), and in the two families with the most abundant putative partials, alignments were consistent with a compact structural domain (e.g. Figure 4B). These sequences, as well as some bounded sequences, were used to identify Pfam27 domains that have been divided into smaller structural units by the CATH structure database [14] (Table 2).

**Table 2:** **RPD2 partial domains corresponding to CATH structural domains**

**Family**	**Pfam**	**CATH**	**CATH length**	**Accession**
**(Pfam)**	**length**	**class**	**(1st to 3rd quartile)**	**UniProt/PDB**
PF00079	372	3.30.497.10	109 (54–172)	Q7TP87/1YXA |A
PF00118	485	3.50.7.10	177 (181–181)	F0YNJ0/1A6D |B
		3.30.260.10	47 (38–56)	
		1.10.560.10	125 (115–134)	
PF00285	356	1.10.230.10	109 (104–115)	Q98FC2/2H12 |A
PF00316	325	3.30.540.10	179 (191–192)	P00637/1BK4 |A
		3.40.190.80	132 (132–135)	
PF00476	383	1.20.1060.10	126 (115-182)	A2WDN6/1KFS |A
PF00710	313	3.40.50.1170	214 (214–216)	C7ZQZ8/1HFJ |A
PF01544	292	3.30.460.20	149 (147–151)	D2C657/2BBJ |A
		1.20.58.340	76 (76–76)^a^	
		1.20.58.340	118 (118–118)	
PF01571	211	3.30.70.1400	88 (87–88)	Q83E96/1NRK |A
PF03441	277	1.25.40.80	90 (85–88)	Q4V935/1TEZ |A
PF03598	412	3.30.1650.10	173 (155–178)	B8FZT4/3GIT |A
		3.40.970.20	133 (129–146)	
		3.40.1470.10	93 (89–98)	
PF03917	370	3.30.1490.80	28 (12–45)	Q1RL06/2HGS |A
		3.30.470.20	166 (135–193)	
		1.10.1080.10	41 (33–47)	

^a^The CATH:1.20.58.340 domain is annotated to two adjacent locations covered by PF01544 in [PDB:2BBJ |A].

### Some partial domains result from shorter structural domains

To identify compact sub-domains that might account for RPD2 partial annotations, we compared bounded and candidate partials from the unbounded searches to the sequences in the PDB structure collection [15]. From the 57 RPD2 Pfam27 families that appeared to have a significant number of bounded or unbounded partial domains, we identified 11 that contain multiple CATH structural domains (Table 2) that are also annotated by VAST+ [16]. Table 2 summarizes the clearest examples of structurally compact partial RPD2 Pfam domains.

Many of the examples in Table 2 are straightforward; a Pfam domain aligns with a structure containing multiple CATH structural domains, and each CATH domain is a contiguous sequence. For example, [UniProt:Q98FC2] appears to largely contain a single unbounded partial domain from the C-terminal half of the [Pfam:PF00285] (citrate synthase) model (Additional file 2: Figure S1). But the alignment of [UniProt:Q98FC2] with [PDB:2H12 |A] shows that the structure comprises two CATH domains, the shorter of which (1.10.230.10) is homologous to the C-terminal portion of [Pfam:PF00285]. Figure 4 highlights the structurally compact region of [PDB:2H12 |A] that corresponds to the [Pfam:PF00285] partial domain.

There are 56 PDB structures containing the CATH:1.10.230.10 domain in the current version of CATH, with a mean length of 109 residues and the first and third quartiles ranging from 104 to 115. Thus, the CATH:1.10.230.10 domain is much shorter than the 356 match states of the [Pfam:PF00285] domain, and may explain the Pfam partial domain. In other cases, e.g., [Pfam:PF00118] (TCP-1/cpn60 chaperonin)/[UniProt:F0YNJ0] vs [PDB:1A6D |B], the Pfam domain aligns with portions of structures with multiple CATH domains, some of which are discontinuous in sequence.

In addition to structural partials that could be identified by comparing Pfam and CATH domain annotations, we examined the most abundant putative partials, including [Pfam:PF00852] (histone deacetylase, Table 1). Searches with 142 projected domains from [Pfam:PF00852] against sequences from PDB show two alignment patterns. Half of the alignments were largely full-length alignment, while the other half aligned to only the C-terminal region of the protein, with starting points ranging from 160 to 232 (mean 212). 2NZW chain A, the only homolog to the [Pfam:PF00852] proteins in PDB, is not annotated by CATH. 2NZW |A is annotated by VAST+ [16], which describes a structural domain that aligns with the partial [Pfam:PF00852] domain in [Uniprot:O87156] (Additional file 3: Figure S3). The compact nature of the PF000852 partial is shown in Figure 4B.

For the second most abundant putative partial, [Pfam:PF04734] (neutral/alkaline non-lysomal ceramidase), about 10% of the domains meet the putative partial criterion. Its closest homolog in PDB is 2ZXC, where it aligns with the N-terminal third of the protein, an *α*/*β*/*β*/*α* sandwich in the middle third of the protein structure that is structurally compact (Figure 4C). Although CATH does not annotate this structure, VAST+ assigns a domain that aligns almost exactly with the PF04734 partial (Additional file 3: Figure S3). The third most abundant putative partial family ([Pfam:PF03598]/CO dehydrogenase/acetyl-CoA synthase complex beta subunit) has about 3.5% of domains in this category, and the abundance of putative partial domains decreases gradually to slightly less than 1% of domains at the third quartile. [Pfam:PF03598] is homologous to several proteins with CATH and VAST+ domain annotations, which divide the [Pfam:PF03598] domain into three parts, and one of those parts, CATH 3.30.1650.10, was detected independently in [Uniprot:B8FZT4] (Additional file 4: Figure S2).

Thus, the three most abundant putative partial domains correspond to structurally compact regions based on CATH and VAST+ annotations. Five of the ten most abundant putative partials appear to be structural partials, based on shorter domains found in CATH or VAST. Four of the remaining abundant putative partials Pfam models align a single long CATH or VAST+ domain. In one case ([Pfam:PF02738]), the domain appears to align with repeats of the same structural domain.

We compared our hierarchical strategy for identifying evolutionarily mobile structural partials – identification of putative partial Pfam domains followed by confirmation using CATH – with the simpler strategy of looking at CATH domain content in the protein structures annotated by Pfam. Of the 136 Pfam families in RPD2, 107 are mapped in Pfam to PDB structures that are annotated by CATH. We found 64 of those Pfam families map to PDB structures with only one CATH domain, so they cannot be examples of structural partials. In total, 43 RPD2 Pfam families map to PDB structures containing two or more different CATH domains; these Pfam families might be composed of evolutionarily mobile, structurally compact, sub-domains. But only 11 of those 43 Pfam families were confirmed to be both evolutionarily mobile and structurally compact. In 15 cases, either the CATH sub-domain was not consistent with the evolutionarily mobile sequence, or there was inconsistency between the CATH and the VAST annotation. For another 17 Pfam families, although CATH annotates multiple structural domains, we found very little evidence for evolutionary mobility (fewer than ten putative partials).

Our PDB/CATH/VAST+ searches found 11 Pfam families comprising shorter CATH and VAST+ structural domains, and another 7 Pfam families that do not have homologs annotated in CATH, but contain multiple VAST+ domains (Table 2, Additional file 2: Figure S1, Additional file 4: Figure S2, Additional file 3: Figure S3). The combination of evolutionary mobility (the putative partial domain in different sequence contexts) and structural compactness suggests that these Pfam domains could be subdivided into shorter, independent domains.

## Conclusions

We have tested the hypothesis that Pfam27 domains are largely structurally compact protein building blocks by characterizing the 10% of Pfam domains in the RPD2 database that appear to be shorter than <50% of the characteristic domain length (the Pfam model length). RPD2 protein domains are representative of Pfam27 since they have a distribution of partial domain annotation lengths that is almost indistinguishable from all Pfam27 domains with model lengths greater than 200 match states (Figure 1). However, RPD2 reduces the extreme differences in domain abundance and model length variation. We believe that the RPD2 family subset accurately represents partial domains in Pfam27.

Our results suggest that, with a small number of exceptions, Pfam27 domains are compact structural building blocks. Only about 15% of the Pfam domains in RPD2 appear to have genuine structurally compact, evolutionarily mobile partial domains, suggesting that most Pfam domains do not comprise smaller structural units. Indeed, most apparent partial domains are likely to be alignment, annotation, and sequence assembly artifacts, rather than smaller sub-domains.

The distributions of the three types of candidate partial domains – split domains, and bounded and unbounded partials – differ widely for the different Pfam families in RPD2 (Table 1).

Partial domain annotations can be counted in two ways – by the number of sequences containing the partial or the number of partial domains – but the distribution of partial annotation counts is similar for both measures. About 60% of candidate partial domains are split domains, including the family with the largest number of candidate partials, [Pfam:PF00209] (sodium:neurotransmitter symporter), where 77.8% of the domain annotations are partial, but 72.5% of those partials are split domains. The family-specific nature of partial domains is illustrated by the difference between the extremes (minimum and maximum in Table 1) and the quartiles. There is no partial type that is found in every Pfam family (the minimum is always 0.00), and the maximum percentage is usually tenfold higher than the third quartile percentage. Moreover, the Pfam family with the largest fraction of unbounded partials ([Pfam:PF04734]) is not the same as the one with the largest number of putative unbounded partials ([Pfam:PF00852]). These differences in the types and frequencies of different partial domains reflect interactions between the sensitivity of the HMM domain model, the distribution of domains at different evolutionary distances, and the sequence sampling of the databases used to construct the models.

Partial domain annotations are well recognized in Pfam [10]; they are represented as jagged edges in Pfam’s graphical presentation. But, because the model_start/model_end information is available only through Pfam’s XML or MySQL interfaces, the graphical Pfam presentation makes it difficult to distinguish split domains (Figure 2B) from a repeated set of N- or C-terminal partial domains. Mapping the domain to the HMM allows us to infer that 60% of 50% partials can be made complete by combining split domain partials.

About one sixth of candidate partial domains cannot be extended to produce larger regions, because they are bounded by the end of the protein or by other non-homologous domains (Figure 2C). While some of these bounded partial domains are legitimate partials, some bounded partial domains are likely to be protein assembly artifacts, while others may be alternatively spliced isoforms. Light and Elofsson [17] examined how alternatively spliced isoforms can produce structurally incomplete, non-functional proteins. Indeed, of the 114 human proteins with bounded domains in RPD2, only three, [UniProt:A8MTL9] ([Pfam:PF00079], serpin), [UniProt:Q5T2L2] ([Pfam:PF00248], aldo-keto reductase), and [UniProt:P0C7U1] ([Pfam:PF04734]), were both annotated as reviewed and had an Ensembl gene model. Both [Pfam:PF00079] and [Pfam:PF04734] appear to contain genuine partials (Table 2 and discussion below), and the gene encoding [UniProt:Q5T2L2] ([Ensembl:ENSG00000264006]) is annotated to produce two alternative transcripts with longer coding sequences, both of which are annotated as CDS (CoDing Sequence) incomplete.

About one quarter of our candidate partials map to the Pfam model in a way that leaves room for a much more complete Pfam domain (unbounded partial, Figure 2D). Searches with those extended sequences show that most of them produce long alignments; thus, those domain annotations are partial because of the inability of the Pfam model to capture the entire homologous region for very distant domain homologs (Figure 3). However, about 30% of these extended sequences produced short alignments, suggesting an alternative sequence context (putative partials), and in the two families with the most abundant putative partials, alignments were consistent with a compact structural domain (e.g., Figure 4B). These sequences, as well as some bounded sequences, were used to identify Pfam27 domains that have been divided into smaller structural units by CATH (Table 2). These searches found 11 Pfam families annotated to contain multiple CATH and VAST+ structural domains. Examination of VAST+ domain annotations on RPD2:PDB homologs revealed seven additional structural partials that were not annotated by CATH, including the two most abundant putative partial Pfam families, [Pfam:PF00852] and [Pfam:PF04734].

Structural compactness is not sufficient by itself to explain evolutionarily mobile structural partials. There are many examples of compact structural domains in CATH that do not exist in isolation and are not evolutionarily mobile. For example, CATH annotates two structurally similar half-domains (CATH:2.40.10.10) on human trypsin-1 ([UniprotKB:P07477]), while Pfam annotates a single trypsin domain. Since no proteins contain a single CATH half-domain, we would not consider it a structural partial, and we have excluded proteins with repeated structural domains from the list of candidate structural partials in Table 2 and Additional file 2: Figure S1, Additional file 4: Figure S2, and Additional file 3: Figure S3. To be considered a structural partial, a domain must have a compact structural domain and be found in different sequence contexts.

We emphasize that these are conservative estimates. Our 18 examples of structural partials excluded cases where CATH and VAST+ disagreed, and other cases where the partial domain topologies annotated on the structure were intermingled because different parts of the same structural domain were assigned to non-contiguous regions of the sequence domain annotated by Pfam. However, because the VAST+ structural annotations do not include the domain-based homology classification provided by CATH, we have less confidence that VAST+ domains can be found in different structural contexts. We are very confident, however, that most of the candidate partials, and about half of the putative partials, are not genuine structural partials. They are more likely the result of sequence annotation and assembly errors.

In a companion paper, Prakash and Bateman describe a second mechanism that can produce partial domains in proteins: domain atrophy [18]. Prakash and Bateman avoid some of the partial domain artifacts that we encountered by focusing on bounded partials in proteins with UniProt evidence code 1, thus avoiding gene annotation errors. Both our approaches are conservative. Some of the putative and bounded partials that we found but were not supported by multiple CATH domains may be examples of domain atrophy, and some partials initially ascribed to domain atrophy were later understood to be structural partials where a Pfam domain comprises multiple structural domains.

We suggest that genuine structural partials can be inferred based on two criteria: (1) evolutionary mobility, where the same domain is found in different protein contexts, and (2) structural compactness. However, all structurally compact domains are probably not evolutionarily mobile and there are examples of evolutionarily mobile domains that are not structurally compact. Moreover, in the absence of clear structural data, it is difficult to know whether a domain is in a novel protein context, because even the most sensitive sequence-based comparisons can fail to detect structural homologs. The novel-context question is muddied further by the possibility that some protein sequences were inferred from misassembled genes and genomes. This study shows that most of the candidates we initially characterized as partials are artifacts of partial alignment, splice isoforms, and incorrectly assembled proteins.

Accurate domain identification and boundary characterization can dramatically improve protein annotation [4,5]. Incomplete domain alignments can be detected with reverse sequence searches rather than an HMM alignment. Partial domains in incorrectly assembled proteins present a greater challenge, because tracing a protein back to its original gene model can be time-consuming. It was reassuring to find that the two reviewed human proteins with bounded domains that are complete in Ensembl probably contain structural partials. Our results suggest that gene models and protein predictions that produce partial domains should be reviewed carefully; it is likely that many of those gene models and proteins are incomplete. Conversely, the incorporation of protein domain models should improve current gene annotation strategies.

The concept of proteins built from conserved domain building blocks has fundamentally transformed our understanding of protein evolution, folding, and function. However, our ability to identify and accurately bound domain building blocks is hampered by the technical problems inherent in identifying distant homologs and by inaccurate protein sets. A mixture of model-based (HMM and PSSM) and pairwise-alignment methods can provide more reliable domain identification. Our results suggest that partial protein domains should be viewed with suspicion; most protein domains appear to have a characteristic length.

## Methods

### Protein domain sets

Pfam family models, protein sequences, and mappings of curated pfamA domains to protein sequences and domain models were obtained from the MySQL tables distributed with Pfam version 27 [3], without modification. Our dataset was selected from the RPD2 subset of 136 Pfam26 pfamA families. The RPD2 database Pfam families meet three criteria: (1) model length (model length ≥200); (2) diversity (found in at least two of three kingdoms of life) and (3) abundance (examples in ≥100 proteins) as described in [11]. In addition, RPD2 families must come from Pfam clans whose model lengths differ no more than twofold. Pfam clans are a way of representing distant evolutionary relationships in very diverse families for which a single Pfam HMM cannot capture all the members of the family [12]. Because they can contain many different Pfam families, the families in Pfam clans can have a wide range of model lengths, which can complicate the detection of partial domains.

For this study, the RPD2 families and sequences were projected onto the current Pfam27 domain set, causing some Pfam26 sequences no longer in UniProt [19] to be dropped from the dataset. Likewise, domain coordinates and model lengths were updated to Pfam27.

### Identifying partials: domain and model boundaries

Domain boundaries in the RPD2 protein set were assigned using the pfamA_reg_full_significant table in the Pfam27 mySQL distribution, using seq_start and seq_end to determine the coordinates in the protein sequence, and model_start and model_end to determine the mapping of the protein sequence to the domain model. Only pfamA_reg_full_significant domains with the in_full flag set were used in the analysis, and only sequences with the is_fragment field set to zero were used.

The model_length field from the PfamA table was used to determine the full domain length. Partial domains that covered less than 50% of the model_length were identified using the relationship model_end −model_start + 1 <0.5model_length. The number of partial domains changes very little if seq_end and seq_start are used in place of model_end and model_start.

### Sequence similarity searches

Candidate extended domains (unbounded partials) were compared to the RPD2 protein sequence database using the SSEARCH program (version 36.3.6) [20], using the BLOSUM62 scoring matrix with −11/ −1 gap-open/gap-extend penalties. SSEARCH version 36.3.6 allows an alignment score to be subdivided based on domain annotations. Alignment annotation was produced using the pfamA_reg_full_significant table for RPD2 sequences.

Candidate partial domains were compared to the PDB database of sequences with known structures [15], using the CATH protein structure classification database [14] and VAST+ [16] to annotate structural domains. The FASTA web site [21] was used to compare domain annotations produced in alignments of Pfam27 annotated RDP2 sequences with CATH and VAST+ annotated PDB sequences.

### Enrichment analysis

In addition to identifying sequences with partial domains, RPD2 sequences that lacked <50% partial domains were identified. These sequences, and sequences from RPD2 families with more than ten end-bound partials, were divided into eukaryotic and non-eukaryotic groups based on their National Center for Biotechnology Information (NCBI) taxon_id. Eukaryotic sequence enrichment was calculated using the fisher.test() function from the R statistical analysis package. The R qvalue() function was used to estimate the number of families that were significantly enriched for eukaryotic end-bounded partials. We also used the NCBI taxon_id to identify proteins from very well-annotated genomes (human, mouse, and *Drosophila*) and identified the subset of RPD2 proteins that have an Ensembl gene model [22].

## Additional files

Additional file 1
**Distribution of partial domain types for each of the RPD2 Pfam27 families.** The Pfam27 PfamA family accession, total number of domains in RPD2, total number of <50% partials, total number of sequences with at least one < 50% partial, and numbers of split domains (see text), bounded domains, unbounded domains, and putative partial domains is shown for each of the 136 Pfam27 PfamA families in RPD2.

Additional file 2
**Alignments of sequences with RPD2/Pfam27 domains that are found in different structural contexts.** For each RPD2/PfamA family, several Uniprot proteins containing the same PfamA candidate structural partial are shown aligned with the sequence of a protein structure containing multiple CATH or VAST domains. The upper (or lower) solid horizontal line depicts the indicated protein sequence. The boxes above the line represent Pfam27 domain annotations on the sequence, with the model-start and model-end coordinates inside the boxes. The boxes below the second horizontal line show the locations of CATH or VAST domains on the structure used in the alignment.

Additional file 3
**See legend for Additional file** 2 **.**

Additional file 4
**See legend for Additional file** 2 **.**

## Abbreviations

HMM: Hidden Markov model
NCBI: National Center for Biotechnology Information
PSSM: position-specific scoring matrix
RPD2: RefProtDom2
