# Supplementary material for: Most partial domains in proteins are alignment and annotation artifacts
Source: Genome Biol. 2015 May 15;16(1):99. doi: 10.1186/s13059-015-0656-7 (PMC4443539; doi:10.1186/s13059-015-0656-7)
Supplement: Additional file 1 — Distribution of partial domain types for each of the RPD2 Pfam27 families. The Pfam27 PfamA family accession, total number of domains in RPD2, total number of <50% partials, total number of sequences with at least one < 50% partial, and numbers of split domains (see text), bounded domains, unbounded domains, and putative partial domains is shown for each of the 136 Pfam27 PfamA families in RPD2. [file 13059_2015_656_MOESM1_ESM.docx]

Additional Table I.

Domains, partial domains, and partial domain classes in RPD2

| Family | Total domains | Total^1^  < 50% partials | Unique^2^ < 50% partials | Split  domains | Bounded domains | Un-bounded domains | Putative partial |
| --- | --- | --- | --- | --- | --- | --- | --- |
| PF00209 | 5614 | 2670 | 2507 | 2492 | 34 | 144 | 23 |
| PF02738 | 5157 | 2551 | 1125 | 1782 | 563 | 206 | 170 |
| PF00330 | 5911 | 2451 | 1767 | 2316 | 17 | 118 | 5 |
| PF00067 | 5412 | 1910 | 1574 | 638 | 1030 | 242 | 176 |
| PF00374 | 2789 | 1671 | 1292 | 938 | 58 | 675 | 23 |
| PF00346 | 4516 | 1308 | 1294 | 1248 | 34 | 26 | 7 |
| PF03069 | 1264 | 970 | 503 | 935 | 25 | 10 | 7 |
| PF01496 | 1835 | 924 | 670 | 866 | 15 | 43 | 5 |
| PF00318 | 4180 | 800 | 425 | 753 | 32 | 15 | 7 |
| PF00481 | 3493 | 648 | 526 | 461 | 142 | 45 | 44 |
| PF01571 | 4724 | 627 | 540 | 182 | 434 | 11 | 14 |
| PF00082 | 5082 | 625 | 594 | 297 | 243 | 85 | 45 |
| PF01544 | 4868 | 566 | 532 | 114 | 305 | 147 | 57 |
| PF02902 | 1868 | 509 | 487 | 58 | 411 | 40 | 59 |
| PF01425 | 4968 | 500 | 393 | 281 | 86 | 133 | 66 |
| PF00183 | 2823 | 437 | 370 | 218 | 111 | 108 | 61 |
| PF00501 | 6557 | 409 | 349 | 150 | 133 | 126 | 39 |
| PF00136 | 1868 | 378 | 342 | 251 | 67 | 60 | 28 |
| PF00118 | 4881 | 374 | 346 | 102 | 153 | 119 | 134 |
| PF01139 | 1666 | 343 | 323 | 194 | 18 | 131 | 18 |
| PF00079 | 2258 | 330 | 270 | 159 | 43 | 128 | 19 |
| PF00510 | 5015 | 298 | 259 | 210 | 75 | 13 | 8 |
| PF00773 | 4132 | 269 | 261 | 228 | 11 | 30 | 3 |
| PF00351 | 844 | 263 | 143 | 243 | 6 | 14 | 6 |
| PF00230 | 4876 | 260 | 242 | 140 | 59 | 61 | 33 |
| PF03098 | 1118 | 260 | 218 | 201 | 10 | 49 | 5 |
| PF00348 | 4969 | 253 | 238 | 205 | 21 | 27 | 15 |
| PF04734 | 478 | 247 | 227 | 41 | 174 | 32 | 50 |
| PF00454 | 2345 | 240 | 232 | 25 | 196 | 19 | 24 |
| PF00248 | 4888 | 229 | 204 | 71 | 59 | 99 | 28 |
| PF00285 | 4548 | 220 | 215 | 166 | 22 | 32 | 6 |
| PF00141 | 4958 | 218 | 192 | 57 | 91 | 70 | 73 |
| PF00852 | 719 | 208 | 205 | 11 | 179 | 18 | 153 |
| PF00194 | 1524 | 199 | 163 | 85 | 77 | 37 | 33 |
| PF00654 | 4720 | 195 | 169 | 77 | 19 | 99 | 12 |
| PF01142 | 1293 | 172 | 135 | 133 | 27 | 12 | 13 |
| PF01474 | 984 | 169 | 101 | 151 | 1 | 17 | 1 |
| PF00297 | 3484 | 168 | 166 | 5 | 140 | 23 | 16 |
| PF01866 | 601 | 164 | 111 | 132 | 18 | 14 | 12 |
| PF00909 | 3795 | 162 | 139 | 68 | 21 | 73 | 15 |
| PF00476 | 3536 | 161 | 143 | 53 | 84 | 24 | 29 |
| PF00850 | 3234 | 159 | 130 | 77 | 42 | 40 | 20 |
| PF03055 | 1132 | 159 | 125 | 92 | 10 | 57 | 8 |
| PF01255 | 3750 | 152 | 148 | 27 | 58 | 67 | 22 |
| PF02219 | 2799 | 149 | 148 | 8 | 65 | 76 | 5 |
| PF00122 | 4864 | 144 | 135 | 34 | 69 | 41 | 29 |
| PF00557 | 4935 | 143 | 138 | 96 | 33 | 14 | 14 |
| PF01048 | 4874 | 142 | 127 | 30 | 75 | 37 | 13 |
| PF02906 | 1405 | 134 | 119 | 93 | 28 | 13 | 11 |
| PF05649 | 1960 | 134 | 111 | 57 | 28 | 49 | 6 |
| PF01063 | 4871 | 132 | 132 | 0 | 105 | 27 | 8 |
| PF00421 | 1016 | 130 | 120 | 115 | 2 | 13 | 2 |
| PF03441 | 2799 | 129 | 124 | 17 | 74 | 38 | 4 |
| PF00710 | 3312 | 124 | 122 | 6 | 95 | 23 | 42 |
| PF00016 | 2600 | 122 | 106 | 83 | 26 | 13 | 8 |
| PF00199 | 3180 | 111 | 94 | 41 | 32 | 38 | 2 |
| PF01156 | 3428 | 109 | 108 | 38 | 24 | 47 | 16 |
| PF11838 | 1946 | 106 | 93 | 22 | 47 | 37 | 29 |
| PF03936 | 1337 | 104 | 99 | 15 | 47 | 42 | 10 |
| PF01218 | 1372 | 103 | 88 | 87 | 4 | 12 | 4 |
| PF00303 | 2707 | 102 | 100 | 10 | 64 | 28 | 15 |
| PF01915 | 3823 | 101 | 98 | 10 | 55 | 36 | 21 |
| PF13234 | 351 | 101 | 87 | 33 | 66 | 2 | 3 |
| PF01126 | 733 | 100 | 91 | 20 | 68 | 12 | 3 |
| PF03372 | 4638 | 100 | 100 | 4 | 70 | 26 | 29 |
| PF03747 | 1816 | 100 | 99 | 20 | 28 | 52 | 10 |
| PF00115 | 4955 | 99 | 91 | 44 | 9 | 46 | 2 |
| PF08326 | 328 | 96 | 83 | 41 | 41 | 14 | 4 |
| PF03595 | 1673 | 95 | 90 | 14 | 6 | 75 | 5 |
| PF01068 | 2232 | 93 | 84 | 50 | 34 | 9 | 8 |
| PF00148 | 2284 | 86 | 77 | 16 | 44 | 26 | 13 |
| PF02586 | 1499 | 83 | 80 | 10 | 32 | 41 | 21 |
| PF00274 | 980 | 79 | 74 | 47 | 10 | 22 | 9 |
| PF05192 | 4128 | 76 | 76 | 5 | 66 | 5 | 9 |
| PF00657 | 1657 | 75 | 72 | 7 | 56 | 12 | 22 |
| PF02781 | 2886 | 74 | 71 | 41 | 6 | 27 | 5 |
| PF02574 | 2945 | 73 | 67 | 27 | 10 | 36 | 6 |
| PF01179 | 700 | 72 | 62 | 45 | 6 | 21 | 4 |
| PF01137 | 712 | 69 | 60 | 30 | 22 | 17 | 8 |
| PF01702 | 3243 | 68 | 65 | 10 | 35 | 23 | 13 |
| PF00291 | 4887 | 64 | 62 | 11 | 16 | 37 | 5 |
| PF00221 | 2006 | 62 | 59 | 13 | 16 | 33 | 7 |
| PF00368 | 1264 | 61 | 61 | 4 | 48 | 9 | 6 |
| PF00231 | 3303 | 59 | 51 | 15 | 14 | 30 | 12 |
| PF01442 | 1016 | 59 | 46 | 40 | 17 | 2 | 1 |
| PF00223 | 775 | 56 | 45 | 9 | 20 | 27 | 18 |
| PF00344 | 3710 | 56 | 53 | 15 | 8 | 33 | 6 |
| PF01379 | 2548 | 56 | 52 | 37 | 4 | 15 | 3 |
| PF00162 | 3466 | 51 | 42 | 18 | 5 | 28 | 2 |
| PF05139 | 509 | 50 | 41 | 18 | 17 | 15 | 4 |
| PF02333 | 213 | 49 | 34 | 41 | 4 | 4 | 1 |
| PF03917 | 316 | 49 | 44 | 24 | 13 | 12 | 4 |
| PF03972 | 1405 | 49 | 45 | 17 | 8 | 24 | 7 |
| PF01987 | 1217 | 43 | 42 | 7 | 19 | 17 | 3 |
| PF01992 | 801 | 43 | 36 | 19 | 10 | 14 | 3 |
| PF02900 | 1992 | 43 | 38 | 11 | 16 | 16 | 6 |
| PF01937 | 572 | 42 | 38 | 16 | 19 | 7 | 6 |
| PF00494 | 2171 | 38 | 37 | 7 | 16 | 15 | 7 |
| PF00316 | 1657 | 31 | 27 | 12 | 2 | 17 | 2 |
| PF05019 | 321 | 31 | 30 | 10 | 16 | 5 | 5 |
| PF00124 | 1802 | 28 | 24 | 9 | 10 | 9 | 6 |
| PF04227 | 706 | 28 | 24 | 12 | 6 | 10 | 4 |
| PF06032 | 417 | 24 | 18 | 15 | 1 | 8 | 1 |
| PF02676 | 234 | 22 | 17 | 12 | 6 | 4 | 2 |
| PF03253 | 419 | 19 | 19 | 3 | 0 | 16 | 0 |
| PF01887 | 681 | 18 | 18 | 2 | 10 | 6 | 1 |
| PF05426 | 624 | 17 | 17 | 2 | 10 | 5 | 4 |
| PF01129 | 141 | 16 | 16 | 0 | 15 | 1 | 1 |
| PF01341 | 396 | 16 | 13 | 12 | 0 | 4 | 0 |
| PF00161 | 515 | 15 | 15 | 1 | 13 | 1 | 3 |
| PF03945 | 490 | 15 | 15 | 2 | 6 | 7 | 0 |
| PF13660 | 592 | 14 | 13 | 5 | 2 | 7 | 1 |
| PF03598 | 113 | 13 | 13 | 0 | 10 | 3 | 4 |
| PF02431 | 342 | 12 | 11 | 4 | 6 | 2 | 0 |
| PF05719 | 340 | 11 | 11 | 0 | 6 | 5 | 1 |
| PF09754 | 889 | 11 | 11 | 2 | 5 | 4 | 2 |
| PF02436 | 2112 | 9 | 8 | 2 | 2 | 5 | 0 |
| PF02898 | 405 | 9 | 7 | 4 | 0 | 5 | 0 |
| PF05996 | 175 | 9 | 7 | 4 | 1 | 4 | 1 |
| PF12972 | 246 | 9 | 6 | 5 | 0 | 4 | 0 |
| PF02633 | 935 | 8 | 7 | 2 | 3 | 3 | 3 |
| PF03644 | 318 | 8 | 7 | 3 | 2 | 3 | 2 |
| PF01716 | 169 | 7 | 5 | 4 | 2 | 1 | 1 |
| PF03587 | 300 | 7 | 6 | 3 | 1 | 3 | 1 |
| PF09985 | 120 | 7 | 7 | 5 | 0 | 2 | 0 |
| PF01828 | 125 | 5 | 4 | 2 | 0 | 3 | 0 |
| PF04414 | 137 | 5 | 5 | 1 | 2 | 2 | 2 |
| PF02649 | 642 | 4 | 4 | 1 | 1 | 2 | 1 |
| PF11794 | 565 | 4 | 4 | 0 | 1 | 3 | 1 |
| PF01905 | 203 | 3 | 3 | 0 | 1 | 2 | 0 |
| PF02679 | 157 | 3 | 3 | 2 | 1 | 0 | 1 |
| PF07477 | 193 | 3 | 3 | 1 | 0 | 2 | 0 |
| PF08787 | 213 | 2 | 2 | 0 | 2 | 0 | 0 |
| PF14099 | 154 | 2 | 2 | 0 | 1 | 1 | 0 |
| PF02289 | 129 | 1 | 1 | 0 | 0 | 1 | 0 |
| PF07488 | 192 | 1 | 1 | 1 | 0 | 0 | 0 |
| total | 290,148 | 30,961 | 25,116 | 18,624 | 7,250 | 5,087 | 2,156 |

^1^Total number of partial domains.

^2^Number of partial domains in different sequences.
