# Supplementary figures and images for: Most partial domains in proteins are alignment and annotation artifacts
Source: Genome Biol. 2015 May 15;16(1):99. doi: 10.1186/s13059-015-0656-7 (PMC4443539; doi:10.1186/s13059-015-0656-7)

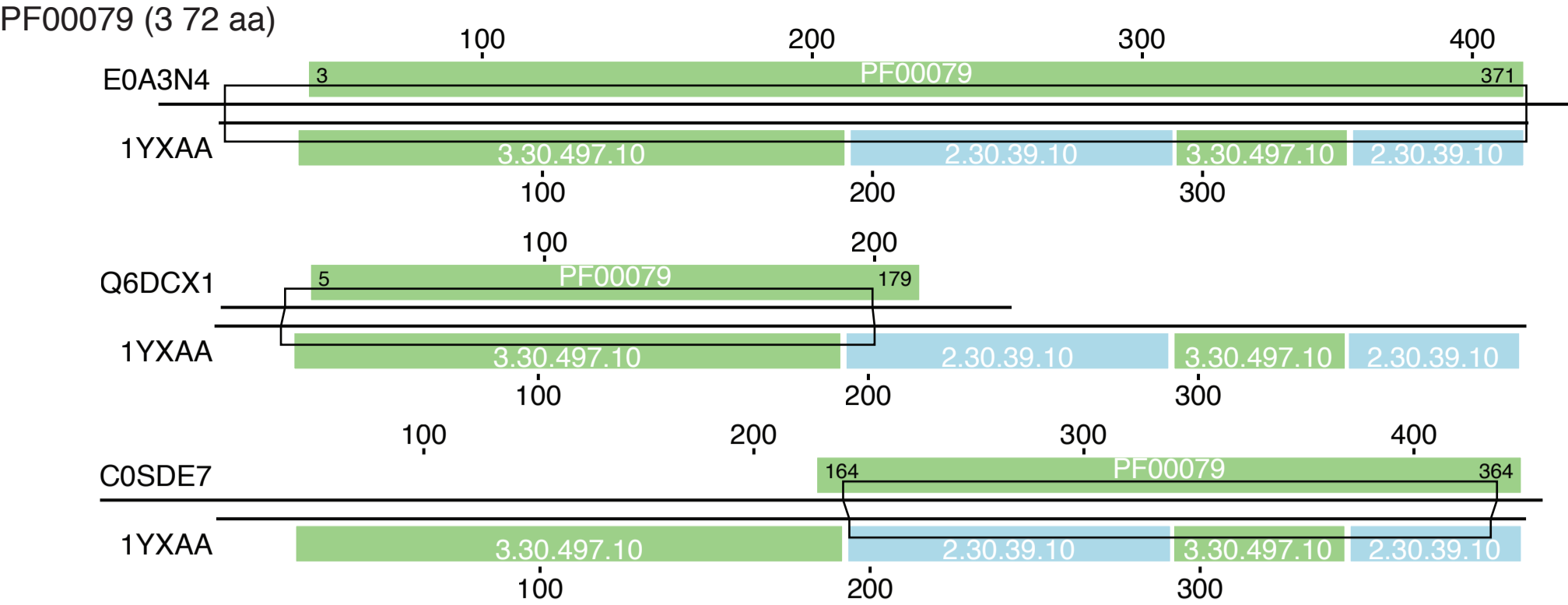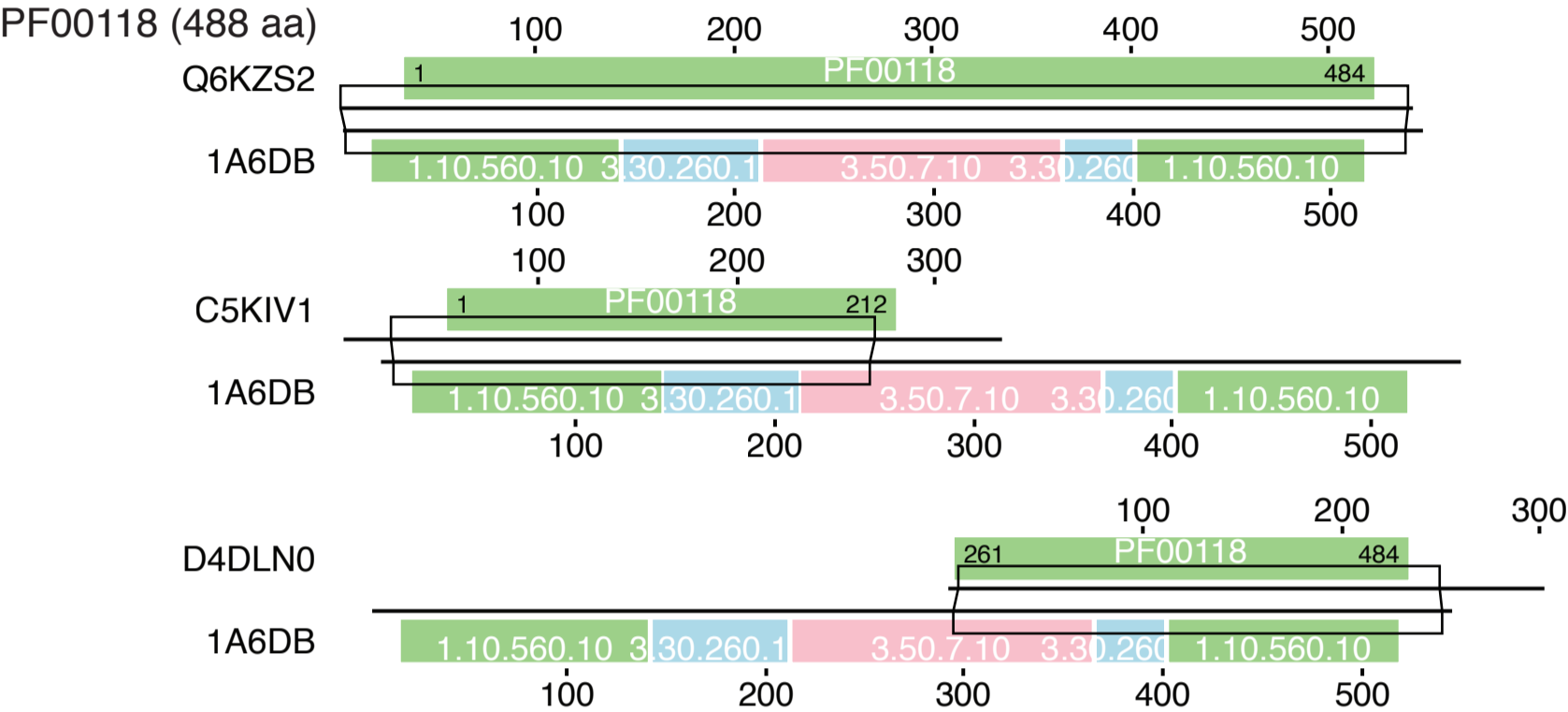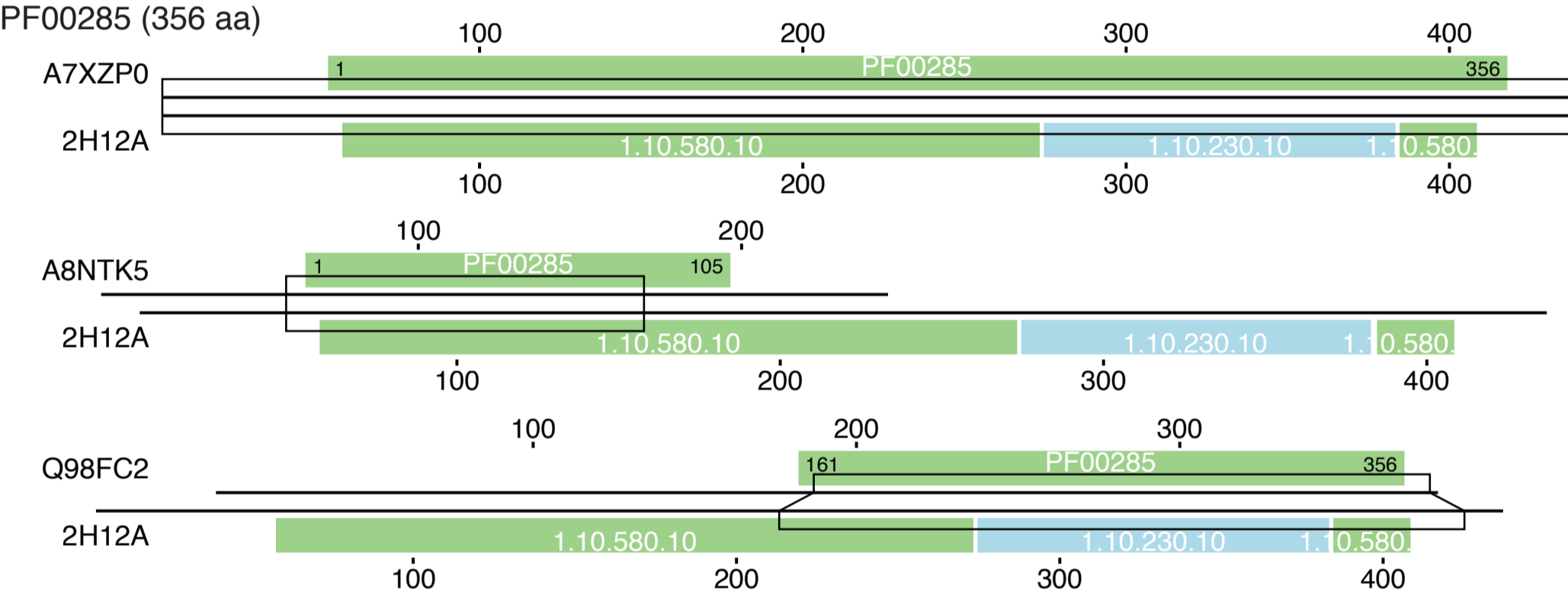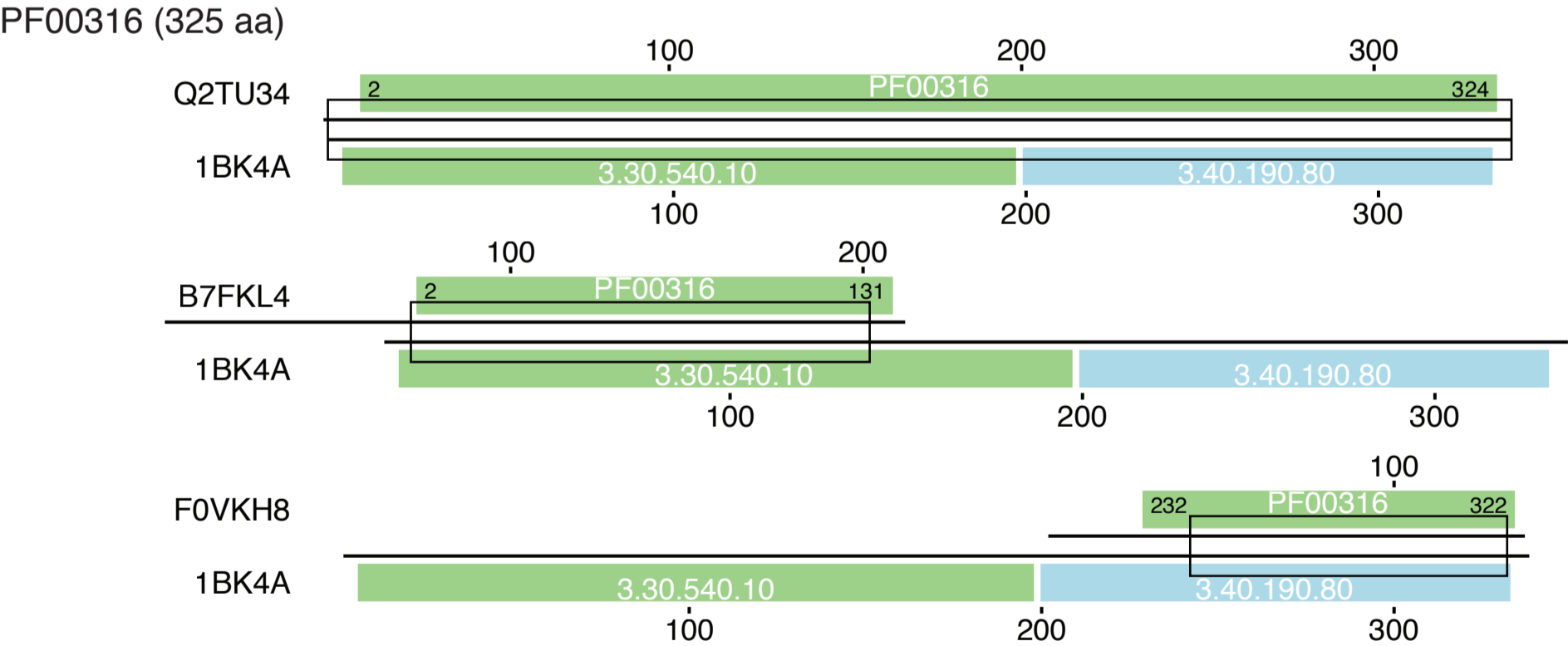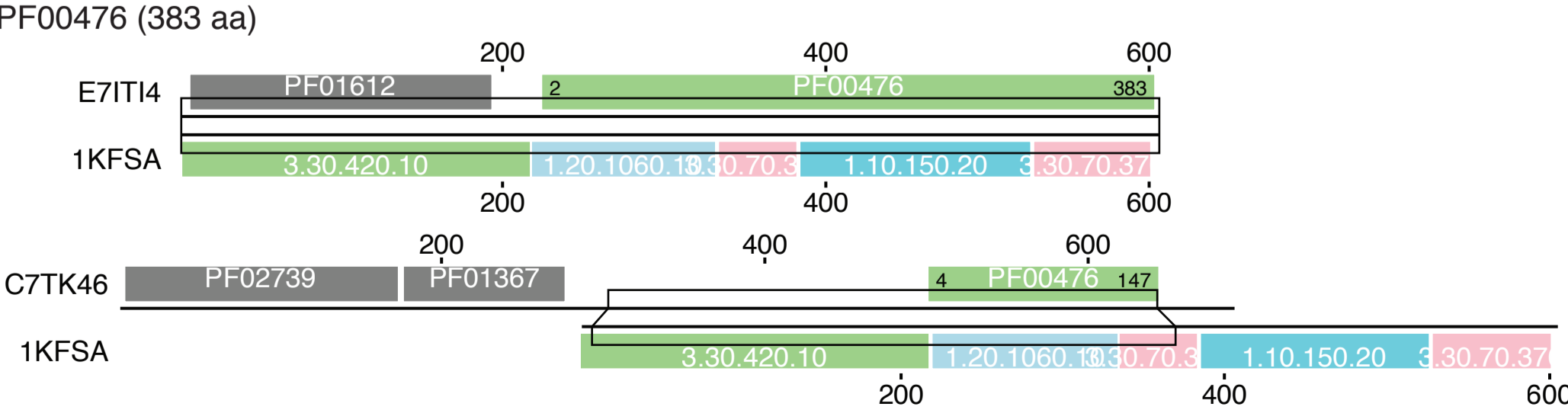

Supplement: Additional file 2 — Alignments of sequences with RPD2/Pfam27 domains that are found in different structural contexts. For each RPD2/PfamA family, several Uniprot proteins containing the same PfamA candidate structural partial are shown aligned with the sequence of a protein structure containing multiple CATH or VAST domains. The upper (or lower) solid horizontal line depicts the indicated protein sequence. The boxes above the line represent Pfam27 domain annotations on the sequence, with the model-start and model-end coordinates inside the boxes. The boxes below the second horizontal line show the locations of CATH or VAST domains on the structure used in the alignment. [file 13059_2015_656_MOESM2_ESM.pdf]

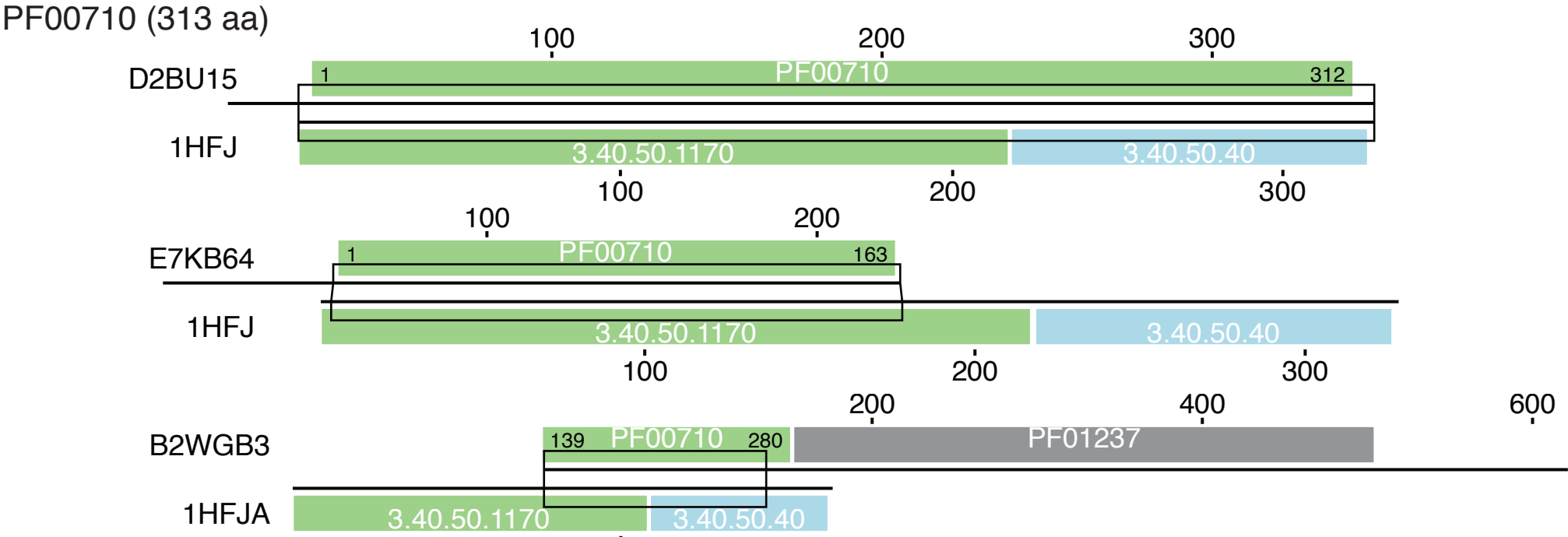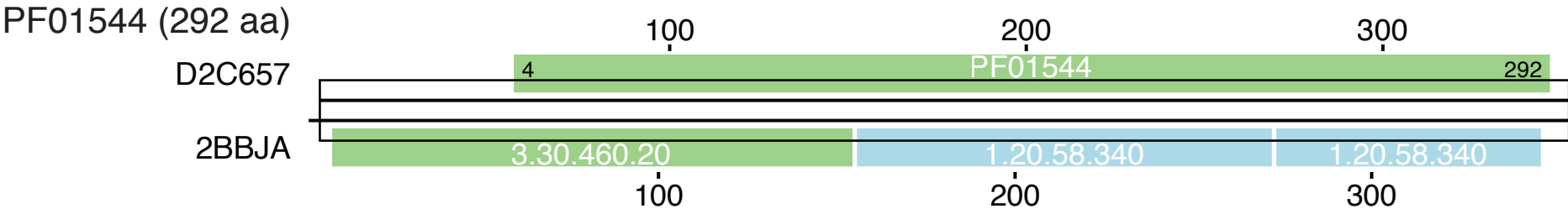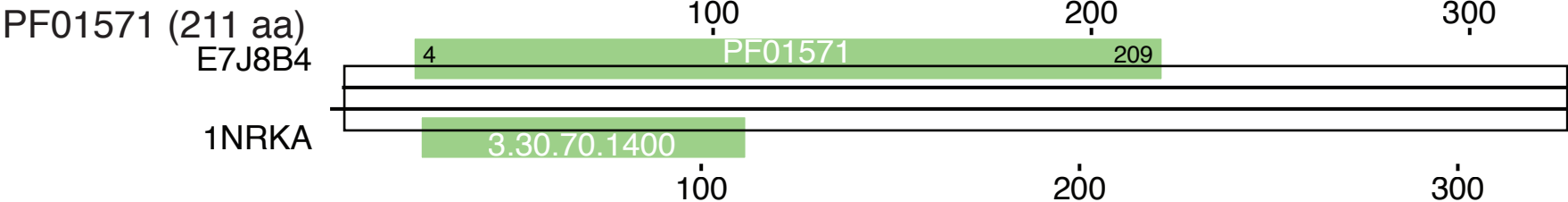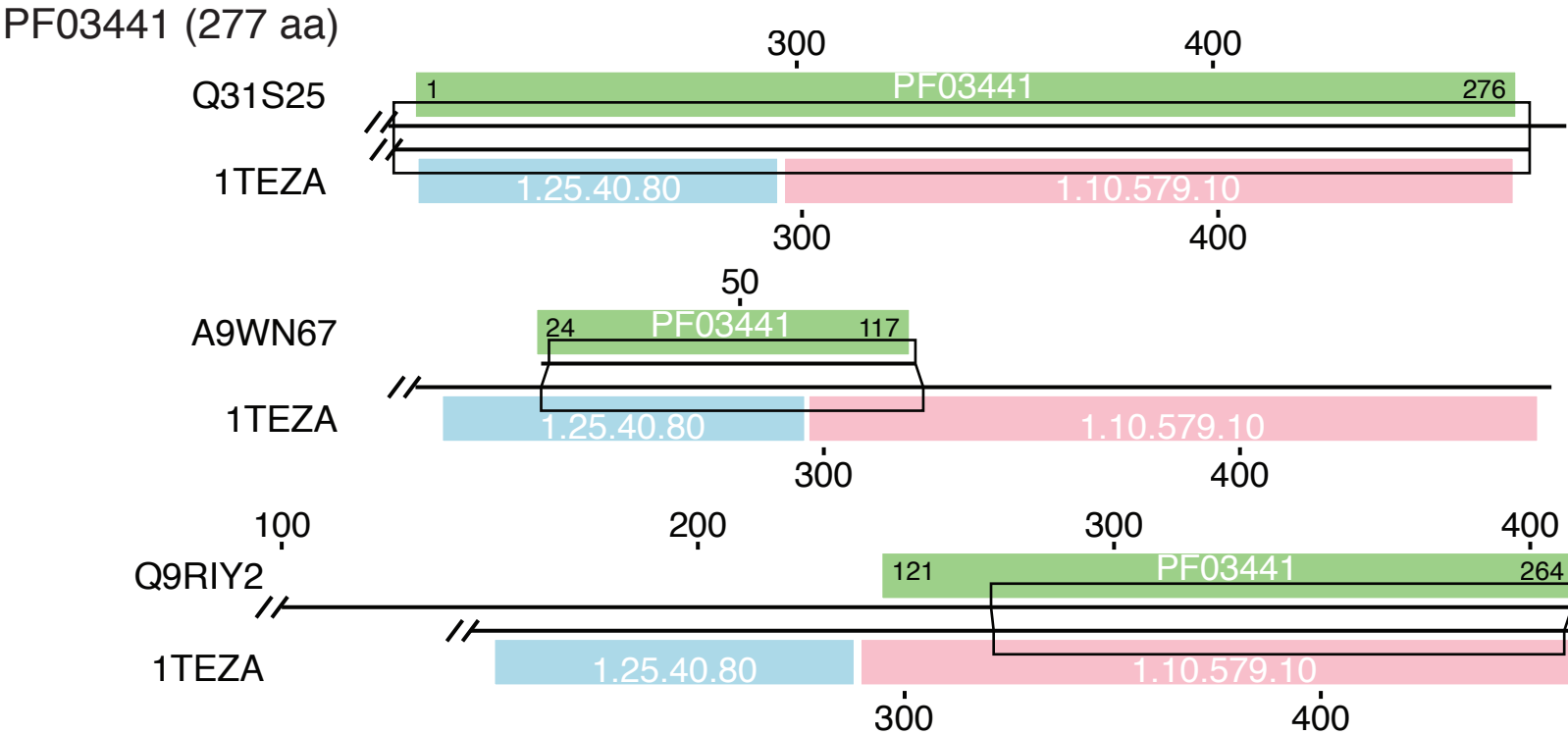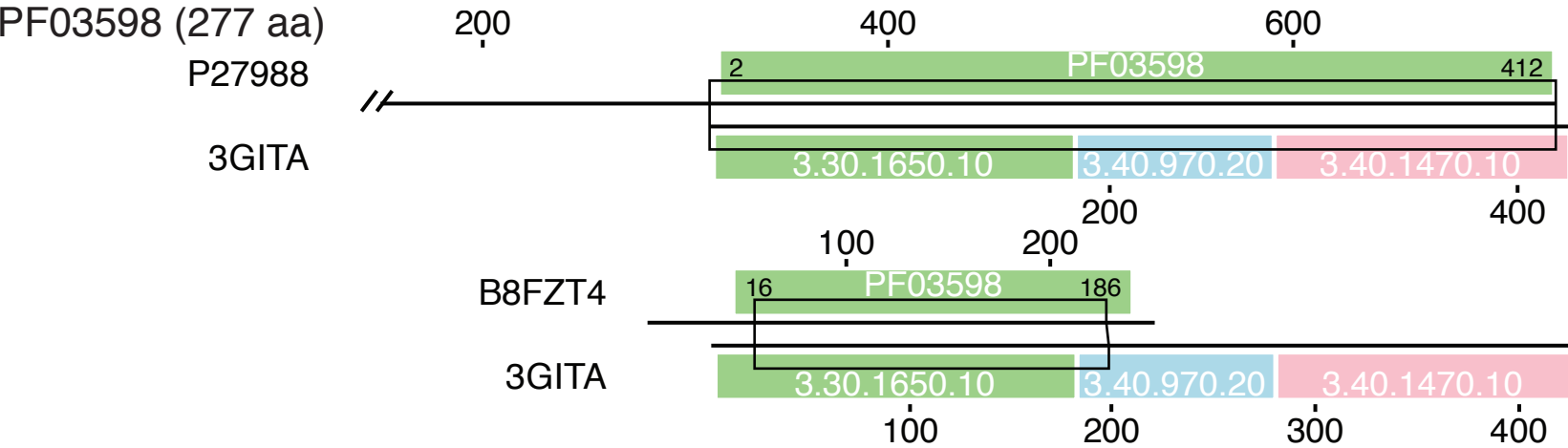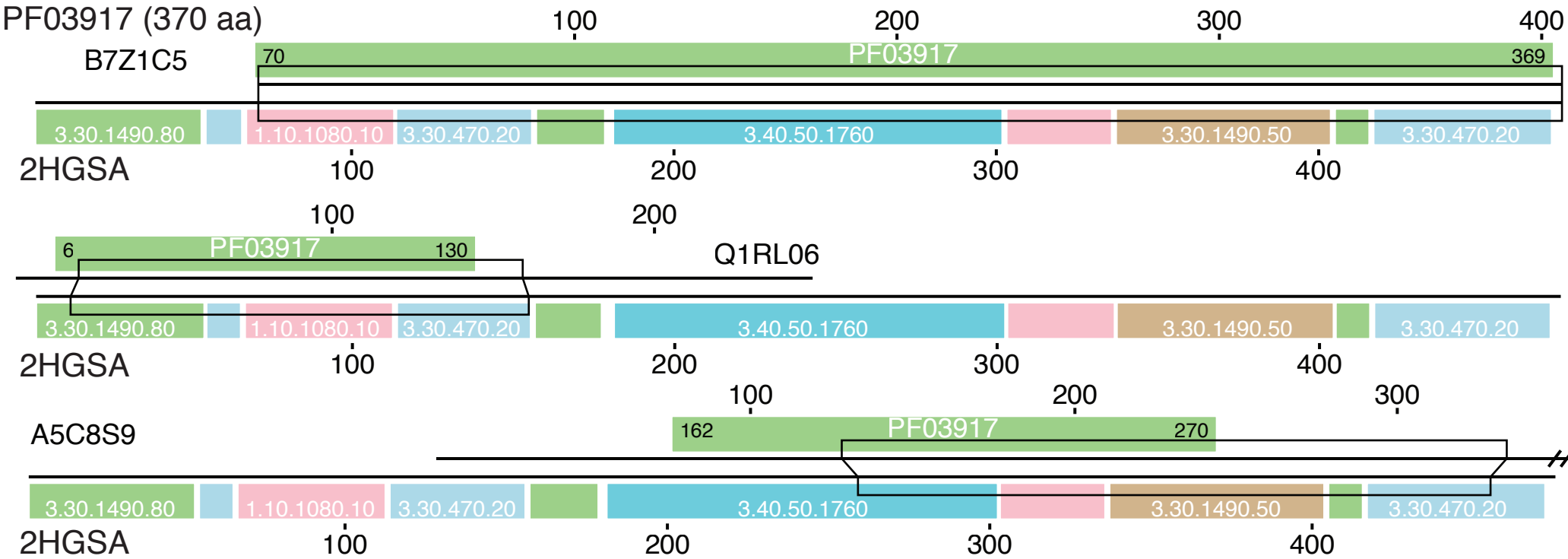

Supplement: Additional file 3 — See legend for Additional file 2 . [file 13059_2015_656_MOESM3_ESM.pdf]

PF00183 (531 aa)

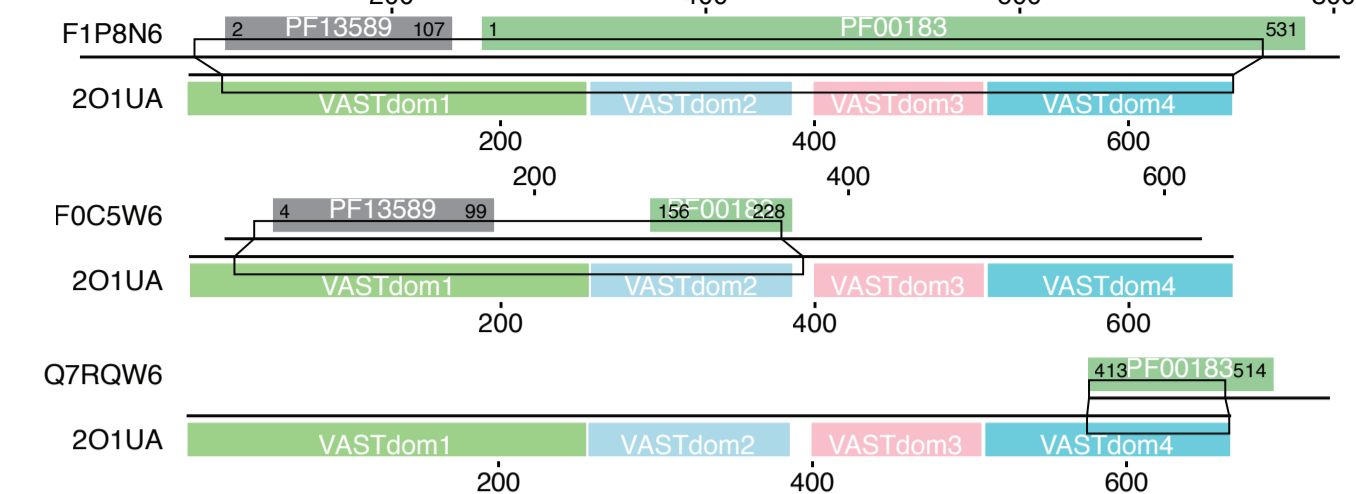

PF00852 (352 aa)

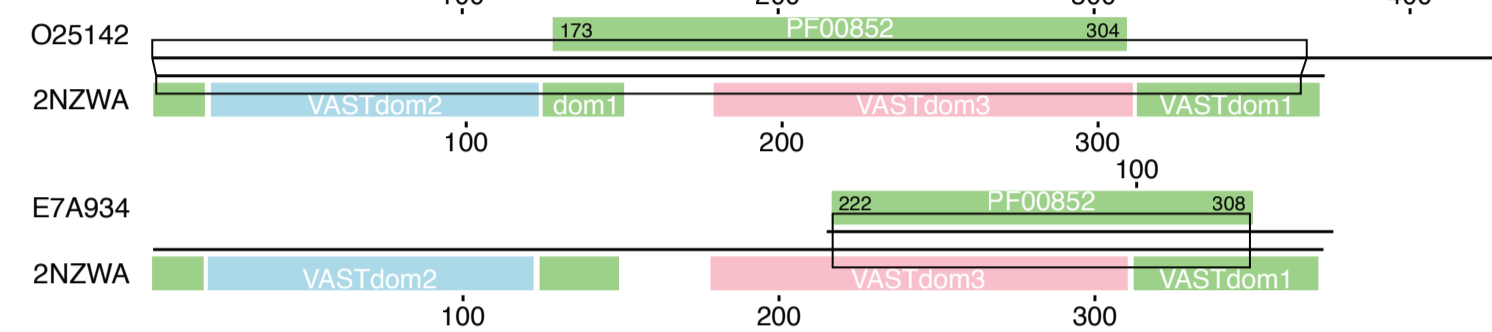

PF01142 (378 aa)

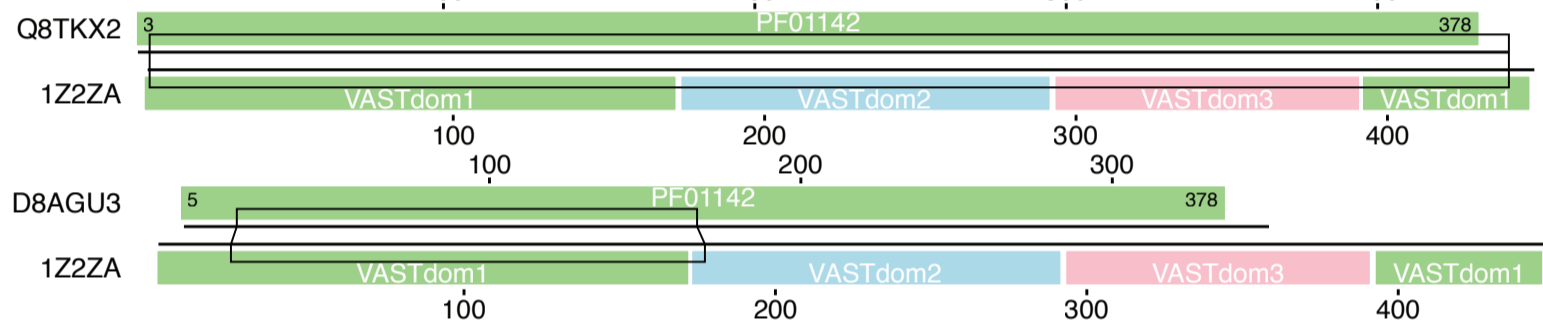

PF03055 (486 aa)

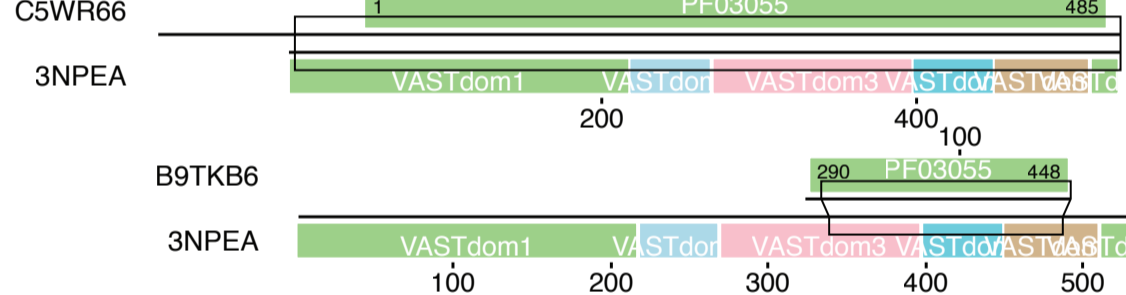

PF03747 (289 aa)

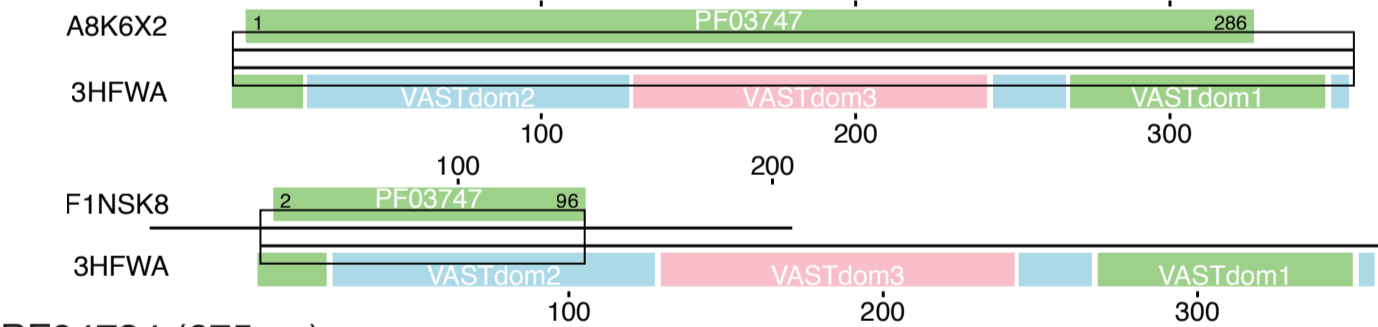

PF04734 (675 aa)

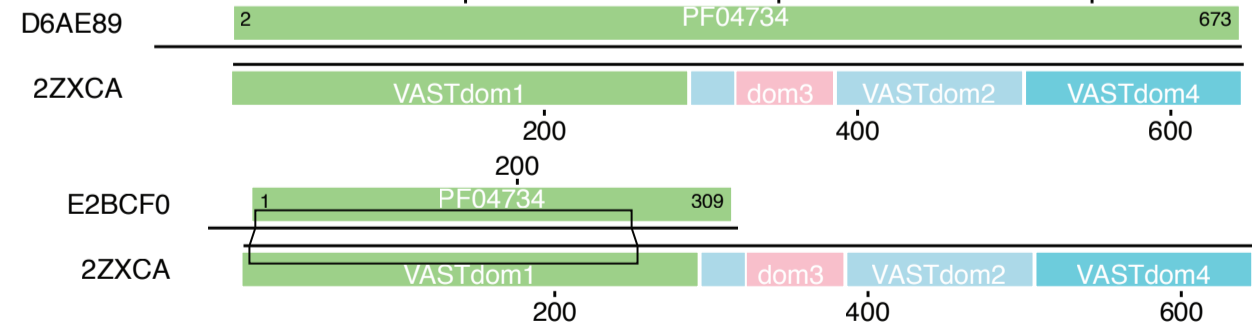

PF11838 (324 aa)

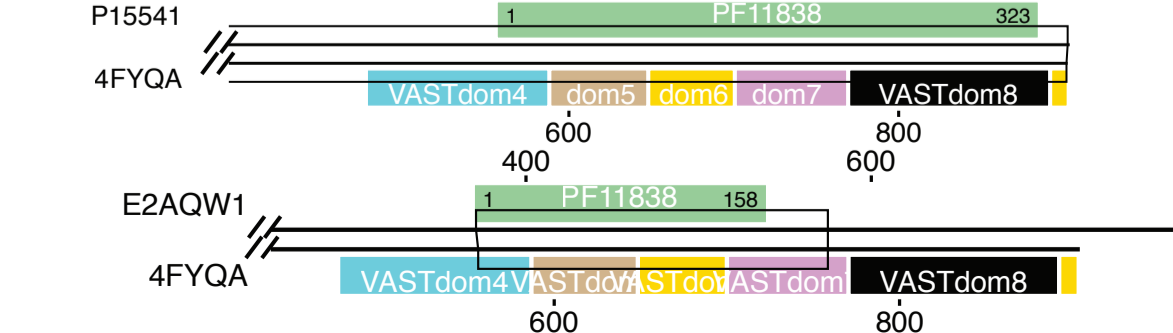

Supplement: Additional file 4 — See legend for Additional file 2 . [file 13059_2015_656_MOESM4_ESM.pdf]
